# Surgical Management of Unusual Biangular Mandibular Fractures

**DOI:** 10.1155/2017/6149838

**Published:** 2017-02-19

**Authors:** Stefan Cocis, Umberto Autorino, Fabio Roccia, Chiara Corio

**Affiliations:** Division of Maxillofacial Surgery, Surgical Science Department, Città Della Salute e Della Scienza Hospital, University of Torino, Torino, Italy

## Abstract

Bilateral mandibular angle fractures, while representing a rarity among mandibular fractures, are a huge challenge of complex management for the maxillofacial surgeon. There are still many open questions regarding the ideal management of such fractures, including the following: the removal of the third molar in the fracture line, the best surgical approach, and the fixation methods. In this report the authors present the case of 40-year-old man presenting with a bilateral mandibular angle fracture referred to the Maxillofacial Surgery Department of Turin. Open reduction and internal fixation has been made for both sides. The left side third molar was removed and the internal fixation was achieved through internal fixation with one miniplate according to Champy's technique and transbuccal access for a 4-hole miniplate at the inferior border of the mandible. Right side third molar was not removed and fixation was achieved through intraoral access and positioning of a 4-hole miniplate along the external ridge according to Champy. An optimal reduction was achieved and a correct occlusion has been restored.

## 1. Introduction 

Mandibular angle fractures are among the most common fractures of the mandible [1]. This has often been related to three main reasons: the presence of the third molar, the thinner cross-sectional area, and the abrupt change in curvature in the angle region [1–3].

Mandibular angle fractures are often isolated or associated with symphyseal or condylar fractures while biangular mandibular fractures (BMF) are instead a rarity [4–6].

Cillo Jr. and Ellis III reported only 33 patients out of 1565 with a BMF over a period of 20 years [5]; Boffano and Roccia observed 8 cases out of 635 over a period of 8 years [6].

In this article the authors report a case of BMF and discuss characteristics and surgical management of this unusual type of injury.

## 2. Case Report

A 40-year-old man was referred to Maxillofacial Surgery Division, Città Della Scienza e Della Salute Hospital, “Molinette”, for mandibular trauma following an assault.

Clinically the patient showed swelling and trismus, tenderness at the mandibular angle, palpation bilaterally, and posttraumatic malocclusion (left posterior precontact).

**Figure 1:**
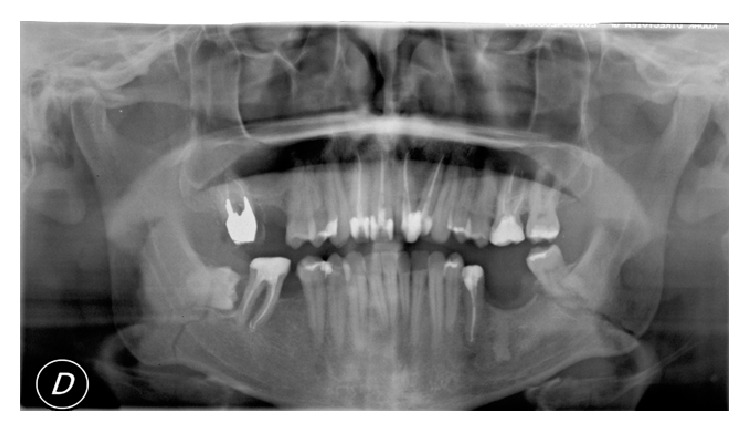
Preoperative panoramic radiograph showing M3 in the fracture lines.

A panoramic radiograph was obtained and revealed a BMF with a mild displacement on the left angle. Moreover it showed the presence of both lower third molars (M3) in the fracture line: left M3 was erupted and presented with a root fracture and right M3 was partially impacted (Figure 1).

After 24 h the patient underwent surgical procedure for open reduction and internal fixation (ORIF) under general anesthesia.

**Figure 2:**
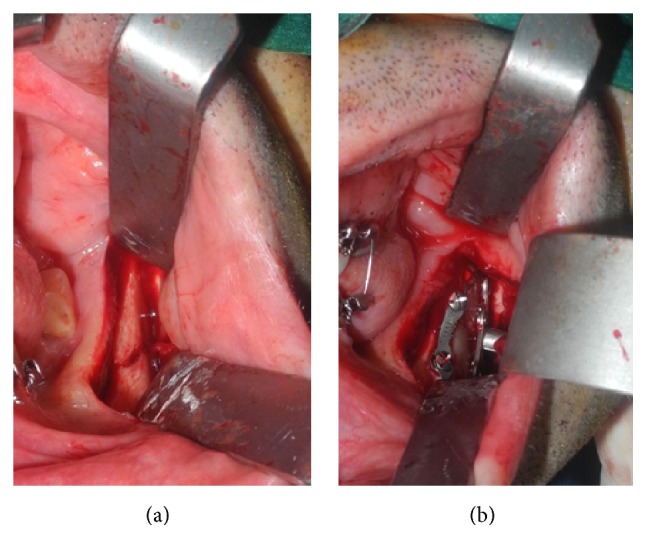
(a) Shows displaced fracture on the left side and (b) shows rigid fixation with a 4-hole miniplate along the external ridge and 4-hole miniplate on the inferior border with the transbuccal trocar.

After the placement of bimaxillary arch bars, each fracture was exposed with intraoral incision. A correct occlusal relationship was obtained after the extraction of left fractured M3 and assured with a temporary intermaxillary fixation (IMF). On this side the fracture was reduced and fixated with a 4-hole with center space noncompression titanium miniplate (Synthes, Michigan, USA) along the external oblique ridge according to Champy et al. [7] (Figures 2(a) and 2(b)).

**Figure 3:**
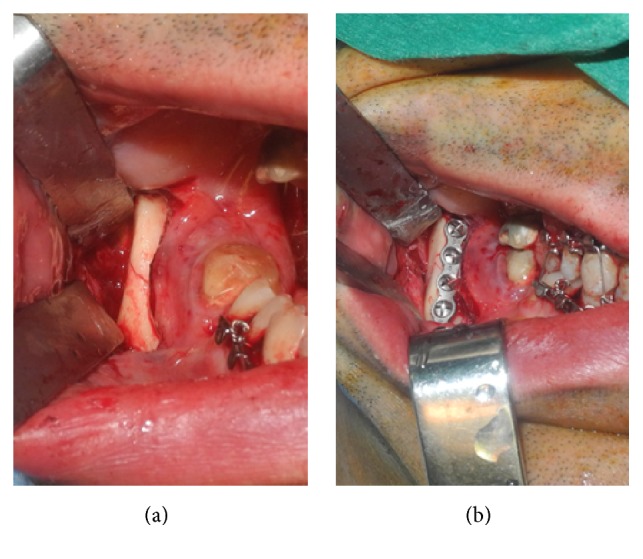
(a) Right side fracture line and (b) showing 4-hole titanium miniplate along external ridge according to Champy.

To assure a rigid fixation on the more displaced side a second 4-hole with center space noncompression titanium miniplate (Synthes, Michigan, USA) was applied on the inferior border via a transbuccal trocar in order to perform the 2.0 mm monocortical screws holes. The right side reduction and fixation was obtained with a single 5-hole noncompression titanium miniplate (Synthes, Michigan, USA) along the external oblique ridge (Figures 3(a) and 3(b)).

After the ORIF the occlusion was checked, the IMF was released, and the incisions were closed with resorbables sutures.

**Figure 4:**
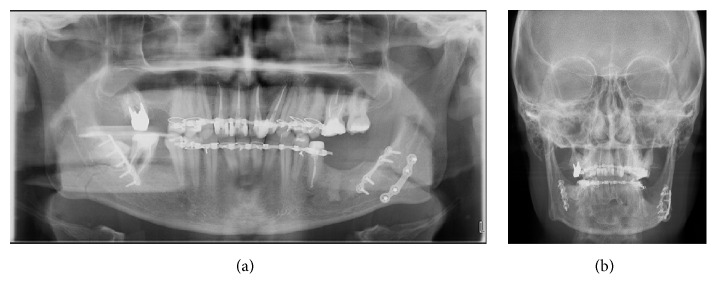
(a) Postoperative panoramic radiograph showing result. (b) Posteroanterior radiograph showing plates configuration and result.

Postoperatively, an antibiotic therapy (intravenous Amoxicillin Clavulanate 2, 2 gr twice a day) was administered for 48 hours. A postoperative panoramic and P-A teleradiographs were obtained one day after the surgery (Figures 4(a) and 4(b)).

The postoperative course was uneventful and the patient was discharged after 2 days with elastic bands IMF for 10 days.

Clinical control 10 days postoperatively showed a normal and stable occlusion and the IMF and arch bars were removed.

No complications were encountered in 1 year's follow-up period.

## 3. Discussion

“The use of one miniplate on the superior border has proved to be the best method with the least complications” [8]; “…both ORIF via an intraoral approach with application of a single monocortical miniplate according to Champy and ORIF via extraoral approach with application of an inferior border plate with at least 2 holes on either side of the fracture line (bicortical) are satisfactory methods of fixation” [9]. These two opposite statements reflect the lack of literature consensus on the treatment of mandibular angle fractures and even less is known about the fixation requirements of bilateral angle fractures. As pointed out by Cillo Jr. and Ellis III [5], they underlined the fact that fixation requirements for bilateral mandibular fractures are not even mentioned in the* Manual of Internal Fixation of the Craniofacial Skeleton* [10] or the* Principles of Internal Fixation of the Craniomaxillofacial Skeleton* [11]. A multitude of treatment options has been proposed for the management of unilateral angle fractures ranging from nonrigid to rigid fixation ranging from large bone plates and compression plates at the lower border to miniplates positioned at the inferior or superior borders and lag screws. These methods have been broadly studied by Ellis III, who has compared eight different modalities of fixation with varying results [1]. Reviewing the latest literature, the most used hardware configuration for mandibular angle fractures result is the Champy's technique [7] and two miniplates technique. Conversely there is little literature about the management of bilateral angle fractures where the surgical challenges are manifold. In a recent study, Cillo Jr. and Ellis III concluded that the bilateral fractures are more unstable than the unilateral variety with the degree of displacement playing an important role in postfixation stability [5]. Moreover in his previous study on combined angle-body or angle-symphysis fractures, Ellis III showed that there is a lower complication rate when rigid fixation is applied to only one of the two fracture sites advocating the use of two miniplates on the more displaced site [12]. This is essentially the treatment option adopted in this case report as suggested by Boffano and Roccia [6] and Ellis III [12]; the most displaced angle was treated by a combined intraoral and transbuccal approach with a rigid two miniplates fixation, whereas the less displaced fracture would receive nonrigid fixation with a single superior border plate via an intraoral access according to Champy et al.'s technique [7]. Both sides showed a correct reduction and no complications were encountered during the follow-up.

Another key point in the surgical management of this type of fractures remains the fate of the M3 in the fracture line. There is more uniformity of view in literature about the fate of M3 as assessed by Bobrowski et al. [13] systematic review and meta-analysis, although this study did not find any difference in postoperative infection rate between the group in which the tooth was removed and the one in which it was conserved. So it seems reasonable to maintain the tooth, unless there is an absolute indication for extraction as suggested by several authors [13–16] who stated that only impacted teeth with cysts or pericoronitis, teeth that prevent a correct reduction, and teeth with fractured roots and with roots exposure should be removed. In this case we proceeded with the extraction of the left M3, which presented with a root fracture, and maintained the right M3, which had no absolute indication for extraction with no complications on either side confirming Bobrowski et al. [13] result that found no statistically significative difference between group that opted for the removal and the group that opted for the maintenance of the M3 in the line of mandibular angle fractures. In conclusion, the management of our patient, consisting of a more rigid fixation of the most displaced angle with two miniplates, a single miniplate placed according to Champy et al. on the less displaced side, and the removal of the third molar presenting fractured root, allowed us to complete a correct and stable reduction with no complications.
